# Peritubular Capillary Rarefaction: An Underappreciated Regulator of CKD Progression

**DOI:** 10.3390/ijms21218255

**Published:** 2020-11-04

**Authors:** Yujiro Kida

**Affiliations:** Department of Nephrology, Takashimadaira Chūō General Hospital, 1-73-1 Takashimadaira, Itabashi City, Tokyo 175-0082, Japan; yujirokida@gmail.com

**Keywords:** chronic kidney disease, endothelial cell, pericyte detachment, peritubular capillary

## Abstract

Peritubular capillary (PTC) rarefaction is commonly detected in chronic kidney disease (CKD) such as hypertensive nephrosclerosis and diabetic nephropathy. Moreover, PTC rarefaction prominently correlates with impaired kidney function and predicts the future development of end-stage renal disease in patients with CKD. However, it is still underappreciated that PTC rarefaction is a pivotal regulator of CKD progression, primarily because the molecular mechanisms of PTC rarefaction have not been well-elucidated. In addition to the established mechanisms (reduced proangiogenic factors and increased anti-angiogenic factors), recent studies discovered significant contribution of the following elements to PTC loss: (1) prompt susceptibility of PTC to injury, (2) impaired proliferation of PTC, (3) apoptosis/senescence of PTC, and (4) pericyte detachment from PTC. Mainly based on the recent and novel findings in basic research and clinical study, this review describes the roles of the above-mentioned elements in PTC loss and focuses on the major factors regulating PTC angiogenesis, the assessment of PTC rarefaction and its surrogate markers, and an overview of the possible therapeutic agents to mitigate PTC rarefaction during CKD progression. PTC rarefaction is not only a prominent histological characteristic of CKD but also a central driving force of CKD progression.

## 1. Background

**Figure 1:**
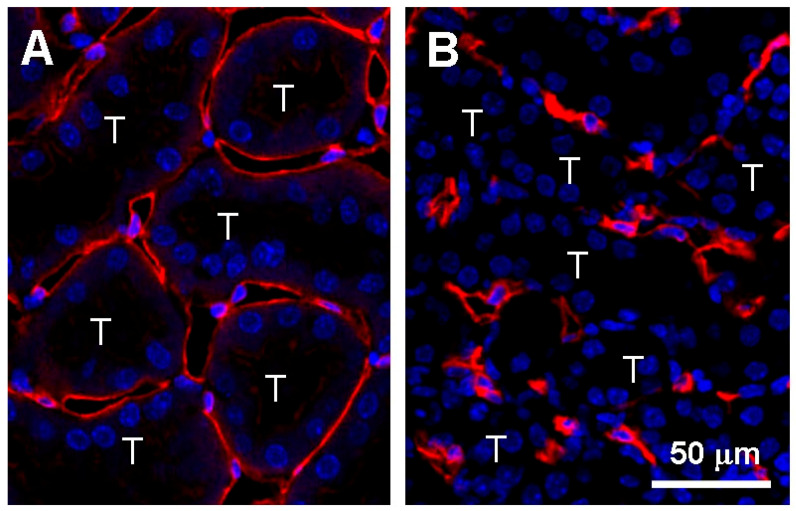
Peritubular capillary rarefaction in the kidney cortex. (**A**) Peritubular capillaries surround tubules in the normal kidneys. (**B**) Peritubular capillaries rarefy after kidney injury, leading to capillary regression. Peritubular capillary endothelial cells are visualized with immunofluorescence staining for CD31 (**red**). Note that many of peritubular capillaries lose their lumina in the injured kidney whereas the capillary lumen is clearly maintained in the normal kidney. Kidney injury is induced by unilateral ureteral obstruction (UUO) in the mouse. T indicates a tubular epithelial compartment. Blue represents nuclei. Scale bar, 50 μm.

Chronic kidney disease (CKD) affects one in every seven adults in the USA, suggesting that 30 million of American adults have CKD [1]. The number of patients with CKD is still growing with the increased prevalence of CKD risk factors such as aging, diabetes, hypertension, and obesity. There is no effective therapy to halt CKD progression to end-stage renal disease (ESRD, the most severe form of CKD). The mortality of patients with ESRD is approximately 15% per year [1]. Moreover, CKD is a major cause of death owing to increasing cardiovascular morbidity [2,3]. One reason for the lack of effective therapies is an incomplete understanding of pathogenesis of CKD. Regardless of initial insults, chronic injuries to the kidney frequently induce tubular atrophy, fibrosis, inflammation and peritubular capillary (PTC) rarefaction (Figure 1). PTC rarefaction is identified not only in diabetic nephropathy and hypertensive kidney diseases, two major causes of CKD [4,5,6], but also in advanced IgA nephropathy [7], congenital nephrotic syndrome [8], lupus nephritis [9], polycystic kidney disease [10,11], and allograft nephropathy [12,13], suggesting that PTC loss is a very common event in patients with CKD. Interestingly, the aging process itself accelerates PTC rarefaction [14]. Although many studies have investigated PTC rarefaction (reviewed in [15,16,17,18]), the molecular mechanisms of PTC rarefaction still remain incompletely elucidated. Recent studies have made major progress in understanding the process of PTC rarefaction. Therefore, based on those new findings, this review describes (1) the novel mechanisms of PTC rarefaction, (2) the major factors regulating PTC angiogenesis, (3) the assessment of PTC rarefaction and its surrogate markers, and (4) possible therapeutic agents to retard PTC rarefaction during CKD progression.

## 2. Anatomy of PTC

**Figure 2:**
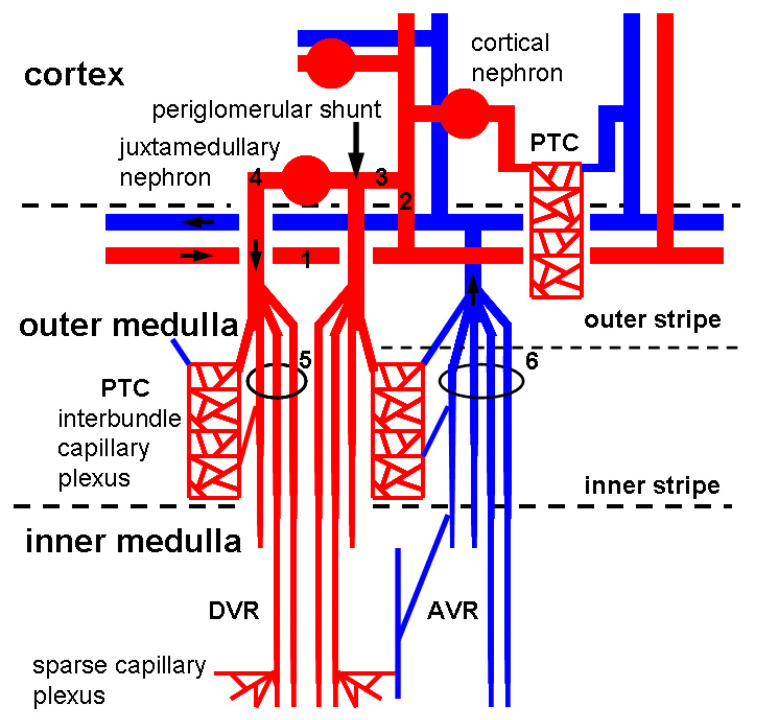
Peritubular microvasculature in the kidney. Arterial blood is delivered to glomerular capillaries in cortical nephrons (90% of total nephrons) and juxtamedullary nephrons (10% of total nephrons) via arcuate artery (labeled as 1), interlobular artery (labeled as 2), and afferent arteriole (labeled as 3). Afferent arterioles occasionally create a periglomerular shunt (indicated with an arrow) but are mostly connected with capillaries in glomeruli without shunting. Efferent arterioles (labeled as 4) in juxtamedullary nephrons construct vascular bundle (labeled as 5) to supply arterial blood for interbundle dense capillary plexus (PTC) and DVR. Venous blood returns to AVR and vascular bundle (labeled as 6) via interbundle dense capillary plexus or sparse capillary plexus. In cortical nephrons, efferent arterioles construct PTC without branches of vasa recta.

**Figure 3:**
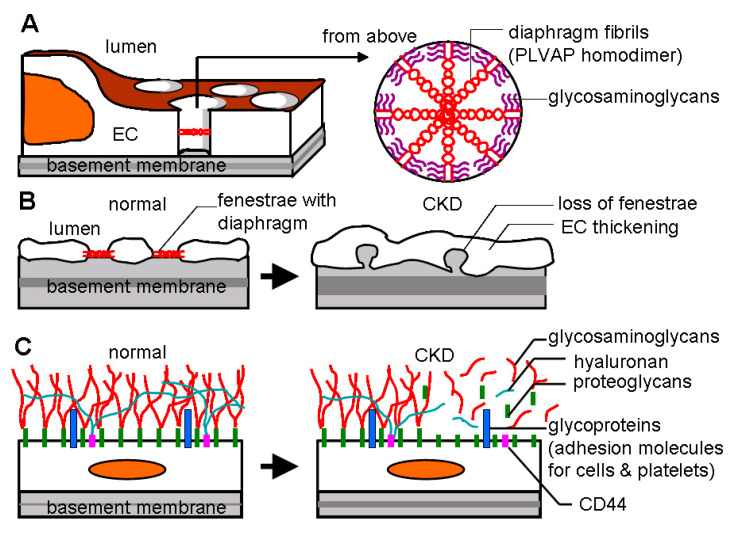
Endothelial fenestrae and glycocalyx. (**A**) Endothelium of PTC including AVR owns fenestrae with 60–80 nm diameters. Each fenestration pore is spanned with multiple units of diaphragm fibrils composed of PLVAP (plasmalemmal-vesicle-associated protein) dimers and glycosaminoglycans. (**B**) In early response to injury, endothelial fenestrae disappear. Endothelial cells (ECs) are subsequently thickening. (**C**) The luminal surface of ECs is coated with glycocalyx consisting of proteoglycans (membrane-bound proteins such as syndecans and glypicans); glycosaminoglycans (long, linear polysaccharides that impart a strong negative charge, including heparan sulfate, chondroitin sulfate, and hyaluronan or hyaluronic acid); and glycoproteins (cell surface receptors such as selectins, integrins, intercellular adhesion molecule [ICAM], and vascular cell adhesion molecule [VCAM]). Injury promotes glycocalyx shedding, which exposes glycoproteins to blood stream, resulting in inflammatory cell adhesion, platelet aggregation, and following coagulation. CD44, one of principle glycoproteins in glycocalyx, anchors hyaluronan chains and stripping of glycocalyx facilitates CD44-leukocyte interaction and leukocyte extravasation. CKD: chronic kidney disease.

Arteriolar blood is delivered to the cortex via a series of large branches of the renal arteries such as interlobar arteries, arcuate arteries (approximate luminal diameter: 70–100 μm in rats [19]), and interlobular arteries (approximate luminal diameter: 40–50 μm in rats [19]). Cortical glomeruli (90% of total glomeruli) and juxtamedullary glomeruli (10% of total glomeruli) branch from interlobular arteries via afferent arterioles (approximate luminal diameter: 21–25 μm in rats [20]). The majority of blood flow reaches the medulla through efferent arterioles (approximate luminal diameter: 19–23 μm in rats [20]) whereas some circumvent glomeruli by a periglomerular shunt (Figure 2). Afferent arterioles are usually larger in diameter than efferent arterioles to increase blood pressure in glomeruli for ultrafiltration to take place. A bundle of glomerular capillaries (approximate luminal diameter: 6–10 μm in rats [21]) forms the efferent arteriole. Efferent arterioles follow one of the two pathways. First, efferent arterioles arise from glomeruli in the mid and outer cortex construct PTC network (approximate luminal diameter: 5–10 μm in rats [22]) running alongside the proximal and distal tubules (cortical nephron) (Figure 2). Second, efferent arterioles arise from juxtamedullary glomeruli construct vasa recta running along the loops of Henle and collecting tubules (juxtamedullary nephron) (Figure 2). Descending vasa recta (DVR, approximate luminal diameter: 13–17 μm in rats [23]) branches into several vessels, penetrating the inner medulla, which construct the sparse inner capillary network (sparse capillary plexus). Venous blood returns to the cortex via ascending vasa recta (AVR, approximate luminal diameter: 18–22 μm in rats [23]) (more details of renal vasculature anatomy are reviewed in [24,25]). Osmotically active solutes (NaCl and urea) move from AVR to medullary interstitium to DVR. This system traps solutes in the interstitium by recycling between AVR and DVR to create the countercurrent exchange for efficient urine concentration. Kidneys lacking AVR do not process the capacity to concentrate urine [26]. A trade-off of countercurrent exchange is that oxygen and nutrients are shunted from DVR to AVR, resulting in profound hypoxia in the inner medulla. Thus, DVR tightly controls perfusion to the outer and inner medulla to avoid the threat of ischemia. Indeed, DVR functions both as an exchanging vessel (capillary) and a resistance vessel (arteriole) [27]. DVR and AVR are not defined as PTCs in this review, because the proximal part of DVRs is an arteriole surrounded by smooth muscle cells [28]. Capillary endothelial cells (ECs) of the glomeruli, PTC network, AVRs, but not DVRs, possess fenestrae that are transcellular pores with 60–80 nm diameters [26,29,30] (Figure 3A,B). Fenestration pores allow water, size- and charge-restricted molecules to pass through. Fenestration pores of PTCs and AVRs display the diaphragmatic structure, but fenestrae of glomerular capillaries lack diaphragms. The diaphragm is composed of multiple radial fibrils and accessory glycosaminoglycans [31] (Figure 3A). Endothelial diaphragm deficient mice demonstrated edematous interstitium in the kidney without any proteinuria and abnormal kidney function, suggesting that PTC diaphragm is involved in transportation of water and proteins in the interstitium [29]. Another prominent structure for EC homeostasis is the glycocalyx, a gel consisting of proteoglycans, glycosaminoglycans, and glycoproteins (approximate height of glycocalyx: 50–100 nm [32]) (Figure 3C). Endothelial glycocalyx covers the luminal surface of ECs and blocks blood coagulation, platelet aggregation, and inflammatory cell adhesion [33].

## 3. Clinical Significance of PTC Rarefaction

While PTC rarefaction was correlated with the severity of fibrosis in patients with CKD [34,35,36], PTC rarefaction was found to be a strong predictor of future ESRD development in CKD patients [37]. Moreover, compared with tubular atrophy, interstitial fibrosis, and inflammatory cell infiltration, the extent of PTC loss reflected glomerular filtration ratio (GFR) most accurately in CKD patients [36]. By using multiple murine CKD models such as cisplatin-induced injury, rhabdomyolysis, and ischemia-reperfusion injury (IRI), another study similarly demonstrated that PTC density correlates with GFR better than fibrosis does [38]. This finding is plausible because a bundle of glomerular capillaries forms the efferent arteriole that is directly connected with PTCs or DVRs without any collateral vessels (Figure 2). As suggested before [39], once PTCs and DVRs disappear or lose their blood flow, perfusion of glomerular capillaries is also dramatically reduced, and vice versa. This idea is supported by a fact that impaired PTC/DVR perfusion causes severe loss of GFR in rat models with congested kidneys [40]. Moreover, the loss of PTC perfusion induces tissue hypoxia that triggers further PTC loss [41]. As perfused PTC number and PTC diameter were inversely correlated well with blood urea nitrogen (BUN) level both in the short term and the long term after severe IRI [42], it is strongly suggested that PTC loss is one of the major contributors to impaired GFR.

## 4. Mechanisms of PTC Rarefaction

### 4.1. Established Major Mechanisms—Loss of Endothelial Survival Factors

Healthy kidneys keep a tight balance between proangiogenic and antiangiogenic factors to prevent unnecessary angiogenesis. However, this balance is disrupted during CKD progression. In response to kidney injury, PTC ECs initially proliferate and subsequently disappear due to endothelial apoptosis [43]. Early proliferation of ECs is supported with intense expression of vascular endothelial growth factor (VEGF)-A, a major and potent proangiogenic factor, in the tubular epithelium compartment. However, expression levels of VEGF-A and its receptor vascular endothelial growth factor receptor-2 (VEGFR2) gradually decline in the later stage, resulting in enhanced EC apoptosis without compensative EC proliferation [43]. Similarly, expression level of angiopoietin (Angpt)-1, another potent proangiogenic factor, is dramatically decreased following kidney injury [44]. Concurrently, antiangiogenic factors such as thrombospondin-1 (TSP-1) and Angpt-2 are strongly induced to antagonize VEGF-A and Angpt-1 signaling [45,46]. Moreover, inflammatory macrophages infiltrate and secrete inflammatory cytokines such as interleukin-1β (IL-1β), IL-6, and tumor necrosis factor-α (TNF-α), all of which block tubular VEGF-A expression [45]. Especially macrophage-derived IL-1β and TNF-α were demonstrated to regress capillary tubes [47]. Deprivation of endothelial survival factors is believed to be a central and major mechanism for PTC rarefaction following chronic kidney injury. As described below, recent research work has discovered other important mechanisms to cause PTC loss during CKD progression.

### 4.2. PTC Endothelium Quickly Responds to CKD Development

A recent ultrastructural study of PTCs revealed that the number of fenestrated pores starts to decrease only 24 h after progressive kidney injury by unilateral ureteral obstruction (UUO) in mice [48]. This time point (24 h post-UUO) was a very early stage in the disease course compared with 5 days post-UUO when significant fibrosis was detectable [49]. Significant PTC rarefaction and endothelial thickening in PTC were detected 3 days and 5 days after UUO, respectively. In the same study, similar fenestration loss and endothelial thickening were observed in other animal CKD models (IRI, Alport mice) and in biopsy samples of CKD patients [48], implying that PTC ECs immediately and continuously react to kidney injury in rodents and humans regardless of the type of insults. The loss of fenestration indicates endothelial activation in glomerular and liver sinusoidal capillaries [50,51,52]. Activated ECs induce heparanase and hyaluronidase that degrade endothelial glycocalyx [53]. Without glycocalyx coverage, ECs become pro-coagulant, pro-thrombotic and pro-adhesive (Figure 3C). For example, the glycoprotein molecule of CD44 on PTC was increased and exposed to the blood stream by glycocalyx stripping after kidney injury, resulting in enhanced neutrophil recruitment/influx, exaggerated inflammation, and PTC loss [54,55] (Figure 3C). Consequently, EC activation leads to impaired blood flow and lowered laminar shear stress on ECs. As shear stress is one of the primary regulators of glycocalyx formation [56], low perfusion of PTCs leads to destabilization and further loss of endothelial glycocalyx. Actually, GFR was inversely correlated with glycocalyx shedding in patients with CKD [57]. Finally, this whole process triggers endothelial apoptosis [58]. These findings suggest that ECs in PTC are promptly activated in response to kidney injury, culminating in PTC loss due to EC apoptosis during CKD progression.

### 4.3. PTC Endothelium Is a Unique Population with Low Proliferative Potential

**Figure 4:**
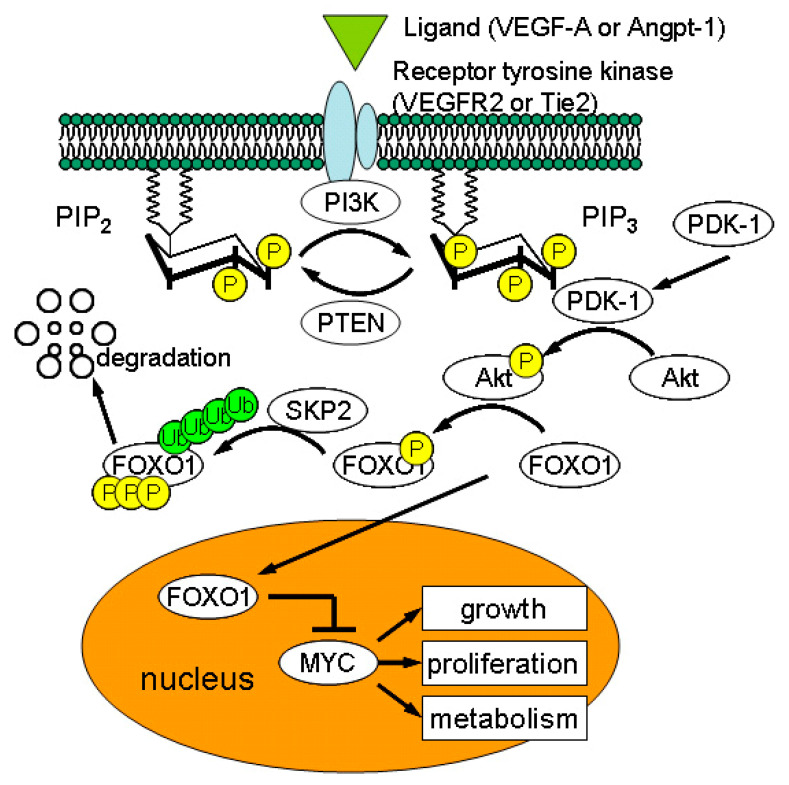
Major signaling pathways for endothelial growth/proliferation. When ligands (vascular endothelial growth factor-A (VEGF-A) or angiopietin-1 (Angpt-1)) bind to receptor tyrosine kinase, activated PI3K (phosphoinositide-3-kinase) converts PIP_2_ (phosphatidylinositol-4,5-biphosphate) to PIP_3_ (phosphatidylinositol-3,4,5-triphosphate) by phosphorylation. Conversely, PTEN (phosphatase and tensin homolog deleted from chromosome ten) turns PIP_3_ into PIP_2_ by dephosphorylation. PIP_3_ is a membrane bound and intracellular messenger that recruits PDK-1 (phosphatidylinositol-dependent kinase 1) and Akt to the plasma membrane. PDK-1 phosphorylates Akt, which, in turn, inhibits nuclear translocation of forkhead box O-1 (FOXO1). As cytoplasmic FOXO1 cannot suppress transcription factor, MYC, enhanced MYC activity leads to increased cellular metabolisms, growth, and proliferation. Phosphorylated FOXO1 is ubiquitinated by SKP2 (S-phase kinase associated protein2 E3 ligase) and is subjected to degradation. When VEGF-A or Angpt-1 is not available, nuclear FOXO1 inhibits MYC activity, limiting endothelial growth/proliferation.

In response to IRI, PTC ECs progressively disappeared with marginal endothelial proliferation [59]. While 0.5–1.0% of PTC ECs were proliferative in normal kidneys, subtle increase in EC proliferation was detected in the early phase of UUO injury [60]. However, the mechanisms underlying low proliferation of PTC ECs have remained unclear. Recent studies identified impaired proliferation of PTC ECs isolated from mouse, rat, and human compared with that of ECs from other organs such as lung and aorta [61,62]. Dang et al. demonstrated that constitutively active phosphatase and tensin homolog (PTEN) suppresses proliferation/growth of kidney microvascular ECs by counteracting phosphoinositide-3-kinase (PI3K)/Akt signaling whereas VEGF-A potentiates PI3K/Akt pathway for capillary growth [62] (Figure 4). In ECs, activated PI3K/Akt signaling prevents nuclear translocation of forkhead box O-1 (FOXO-1) transcription factor, promoting cell cycle progression by enhanced MYC activity [63] (Figure 4). Chemical inhibitor of PTEN not only restored angiogenesis of PTC ECs in vitro but also antagonized PTC rarefaction in IRI in vivo [62]. This finding explains why PTCs are easy to regress after injury.

### 4.4. EC Apoptosis Is a Pivotal Cause of PTC Dropout

CKD progression enhances endothelial expression of active caspase-3 [64] and endothelial apoptosis [43]. In global caspase-3 deficient mice, endothelial apoptosis was significantly reduced post-IRI, resulting in reduced PTC rarefaction [65]. Moreover, loss of caspase-3 lowered hypoxia-inducible factor (HIF)-1α expression following IRI, indicating that preserved PTC network counterbalances tissue hypoxia. Global caspase-3 defect also rescued tubular cells from IRI, probably preserving tubular VEGF-A expression. This would inhibit PTC dropout in mutant mice although VEGF-A level was not measured in this study [65]. Kidney injury increased expression of anti-angiogenic factors such as TSP-1 and endostatin [45,66], both of which induce EC apoptosis by caspase-3 activation [67,68]. Another molecule, CD44, one of endothelial glycocalyx components and induced by kidney injury, strongly enhanced EC apoptosis by caspase-3 activation [69].

### 4.5. Pericyte Detachment Worsens PTC Loss

**Figure 5:**
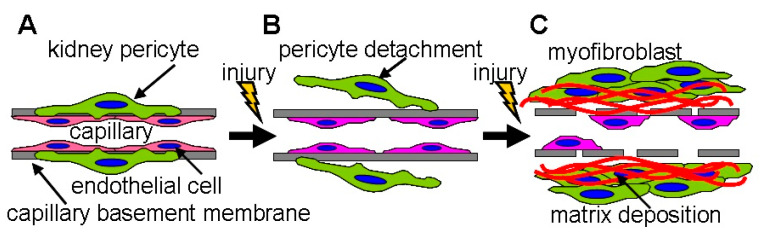
Kidney pericytes are essential to the integrity of peritubular capillaries. (**A**) In peritubular capillaries of the normal kidney, kidney pericytes (**green**) are attached to capillary endothelial cells (**red**) to stabilize capillary tube formation. Pericytes share the capillary basement membrane with endothelial cells. (**B**) In response to injury, kidney pericytes promptly migrate away from the capillary basement membrane, resulting in activation of endothelial cells. (**C**) Following further injury, kidney pericytes differentiate into scar-forming myofibroblasts or activated fibroblasts. These populations synthesize extracellular matrices such as collagens, promoting tissue fibrosis. Capillary endothelial cells are not able to maintain the capillary basement membrane without pericytes. Finally, peritubular capillaries start to disappear due to apoptosis, as myofibroblasts can no longer stabilize capillary tube formation.

Kidney pericytes are closely attached to PTC ECs and maintain the structure and function of PTCs [70,71]. For example, kidney pericytes contact ECs and intensify synthesis of capillary basement membrane to maintain capillary integrity [60]. While kidney pericytes stabilize endothelial tube formation of PTCs in healthy kidneys, pericytes promptly migrate away from PTC following kidney injury, leading to PTC disintegration and rarefaction [60,72] (Figure 5). Images by two photon microscopy revealed that many processes from the cell body of pericytes are attached to PTC in normal kidneys whereas those processes are detached from PTCs and reattached to walls of tubular epithelial cells 3 days after UUO [71]. After injury, pericytes differentiated into two scar-forming populations (α-smooth muscle actin positive myofibroblasts and α-smooth muscle actin negative activated fibroblasts) [73]. Neither fibrotic population could stabilize PTCs after injury [74]. These facts offer the mechanisms by which PTC rarefaction accompanies tissue fibrosis (Figure 5). Kramann et al. identified that Gli1+ cells are a perivascular population of mesenchymal stem cell-like cells [75]. In the kidney, Gli1+ cells functioned as pericytes in normal kidneys, and Gli1+ pericytes were significantly detached from PTCs following IRI [76]. Genetic ablation of Gli1+ pericytes resulted in PTC rarefaction (predominant rarefaction of smaller capillaries whose diameter was less than 7 μm), tissue hypoxia, and transient hypoxic tubular epithelial injury 10 days post-ablation. Interestingly, Gli1+ pericyte ablation induced an inflammatory response with upregulation of renal TNF-α and IL-6 expression. Lemos et al. genetically ablated a forkhead box D-1 (FoxD1) positive population, which induced PTC loss, tubular injury, and albuminuria without acute inflammatory response 3 days after ablation [77]. Loss of FoxD1 was severe enough to sacrifice all mice within 3 days post-treatment. While Gli1+ cells represented a small fraction of kidney pericytes, FoxD1+ population contained pericytes, perivascular fibroblasts, glomerular mesangial cells, podocytes, and vascular smooth muscle cells in the kidney [78]. Both studies reported that pericytes loss causes tubular damage. Injured tubules increased transforming growth factor (TGF)-β expression, promoting pericyte-myofibroblast differentiation and further tissue damage [79]. Studies of pericyte ablation indicate that kidney pericytes are essential for PTC integrity as well as tubular integrity and the loss of pericytes accelerates PTC rarefaction.

## 5. Major Factors Affecting PTC Loss

### 5.1. VEGF-A

VEGF-A is the potent regulator to maintain PTC network. In the adult kidney, VEGF-A is mainly expressed in podocytes and the thick ascending limbs of Henle’s loop and, to a lesser extent, in proximal and distal tubules [80]. Loss of tubular epithelial VEGF-A induced PTC rarefaction even without kidney injury [80]. Moreover, tubular VEGF-A deletion resulted in pronounced polycythemia due to elevated erythropoietin (EPO) production, suggesting that PTC loss induces tissue hypoxia and thereby stimulates renal EPO producing cells [80]. Consistently, expression of VEGF-A was remarkably decreased in rodent CKD models and in CKD patients mainly due to tubular cell atrophy [37,43,45,81]. Conversely, overexpression of VEGF-A in tubular epithelial cells caused overgrowth of PTC ECs that synthesize excess amounts of platelet-derived growth factor (PDGF)-B and TGF-β [82]. This sequence of events resulted in tissue fibrosis because PDGF-B and TGF-β simulates pericyte proliferation and pericyte-myofibroblast transition, respectively [79]. As both loss and overexpression of tubular VEGF-A disorganize PTC architecture, a proper range of tubular VEGF-A expression is indispensable for healthy PTC integrity. Separately, soluble fms-like tyrosine kinase-1 (sFlt-1, soluble VEGFR1) was found to be elevated in patients during CKD progression [83]. sFlt-1 is a truncated form of VEGFR1 and a potent circulating antagonist for VEGF-A. Renal biopsy samples demonstrated that sFlt-1 is expressed in CD68+ histiocytes (a part of macrophage population) and PTC network around sFlt-1+ cells are diminished [84]. In patients receiving cardiac surgery, perioperative low VEGF-A and high sFlt-1 levels in the plasma significantly predicted future development of acute kidney injury (AKI) [85]. In addition, other VEGF-A antagonists such as endostatin [86,87,88] and TSP-1 [89,90,91,92] were increased in patients with CKD, which worsened PTC rarefaction [88,92]. Finally, VEGF-A rich angiogenic macrophages were identified in damaged kidneys from mice and patients [93]. These macrophages were kidney resident, not derived from circulating monocytes, and supported proliferation of PTC ECs. Taken together, reduced tubular VEGF-A expression and increased VEGF-A antagonists cooperatively promote PTC rarefaction during CKD progression.

### 5.2. Angiopoietin/Tie

Angpt-1 and Angpt-2 are another critical angiogenic factors that act on Tie (tyrosine kinase with Ig and EGF homology domains) receptors, Tie1 and Tie2. In the adult kidney, Angpt-1 is expressed in tubular epithelial cells, podocytes, and pericytes, whereas Angpt-2 is detected in ECs, with lower levels in tubular epithelial cells. Tie2 is expressed in glomerular and peritubular ECs in addition to hematopoietic cells. Generally, Angpt-1 binds to Tie2, enhancing EC survival and vascular stabilization, while Angpt-2 competitively inhibits the action of Angpt-1 on Tie2 [94]. In patients with CKD, circulating levels of VEGF-A and Angpt-1 were decreased and those of Angpt-2 were elevated, generating anti-angiogenic environment [46]. Furthermore, elevated Angpt-2 level was a strong predictor of mortality in CKD patients [95]. Inducible and global loss of Angpt-1 accelerated PTC loss and fibrosis only after kidney injury in mice [44], indicating a protective role of Angpt-1 in kidney injury. While Tie2 activation (Tie2 phosphorylation) was reduced by vascular endothelial protein tyrosine phosphatase (VE-PTP) during hypertensive or diabetic kidney injury, inhibition of VE-PTP activated endothelial Tie2 signaling and protected kidneys from such damage [96]. VE-PTP was highly expressed in glomerular and peritubular capillaries and repressed Tie2 activity by its dephosphorylation [96]. Although the role of Tie1 has been ambiguous, recent study showed that Tie1 enhances Angpt-1/Tie2 signaling in ECs [97,98]. However, acute inflammation promoted endothelial Angpt-2 expression and Tie1 ectodomain cleavage, counteracting angiogenesis by impairment of Tie2 signaling [97,98]. Genetic loss of endothelial Tie1 caused capillary regression via markedly enhanced EC apoptosis even if ECs expressed Tie2 [99]. Endothelial overexpression of Angpt-2 induced pericyte detachment from capillary EC, promoting capillary destabilization [100]. Collectively, Angpt-1/Tie2 signaling protects against PTC rarefaction.

### 5.3. HIF

Hypoxia-inducible factor (HIF) is a master regulator of cellular adaptation to hypoxia. HIF is a heterodimeric transcription factor that is composed of HIF-α and HIF-β subunits. HIF-β is constitutively expressed whereas HIF-α expression is tightly controlled by oxygen-dependent degradation. In normoxia, two conserved proline residues of HIF-α are hydroxylated by prolyl hydroxylase domain-containing proteins (PHDs). Hydroxylated HIF-α is detected by the von Hippel-Lindau protein and is subjected to polyubiquitination and following proteasomal degradation. In hypoxia, however, HIF-α escapes from hydroxylation by PHDs and binds to HIF-β. This functional heterodimeric HIF translocates to the nucleus and upregulates the transcription of HIF responsive genes such as EPO and VEGF-A. HIF-α has two major isoforms, HIF-1α and HIF-2α. In the hypoxic kidney, HIF-1α is expressed in tubular and glomerular epithelial cells, whereas HIF-2α is detected in glomerular and peritubular ECs and fibroblasts. Deletion of tubular HIF-1α attenuated tissue fibrosis and macrophage infiltration post-UUO injury [101]. While endothelial-specific HIF-1α inactivation did not show any influence on kidney injury, HIF-2α deletion in the endothelium worsened PTC rarefaction, glomerular capillary loss, albuminuria, and tissue fibrosis following kidney damage [102,103]. Loss of endothelial HIF-2α prolonged inflammatory response [104] and impaired protection against oxidative stresses [105]. Moreover, HIF-2α+ pericytes/fibroblasts were the unique source of renal EPO production [106]. EPO production in kidney pericytes/fibroblasts was exclusively dependent on HIF-2α [107], implying that HIF-2α in pericytes/fibroblasts is protective against anemia and tissue hypoxia. Taken together, renal HIF-1α exaggerates kidney injury, and renal HIF-2α antagonizes PTC loss.

### 5.4. Sirtuin

Silent information regulator two protein (Sirtuin)-1 is a protein with nicotinamide adenine dinucleotide (NAD+)-dependent deacetylase activity and is highly expressed in ECs. Sirtuin-1 antagonizes endothelial cellular senescence (aging process accompanying EC dysfunction) through multiple pathways [108]. ECs stop proliferation after a limited number of doublings. Cessation of cell division induces cell growth arrest, which is termed replicative senescence. Some stresses such as oxidative stress and DNA damage elicit quite similar cell growth arrest in the short term, referred to as stress-induced premature senescence (SIPS). Kidney injury antagonizes PTC angiogenesis by inducing endothelial SIPS. Endothelial Sirtuin-1 prevented capillary loss in the hindlimb model by repressing FOXO1 activity [109]. Furthermore, Sirtuin-1 inactivated p53 by its deacetylation and induced cellular growth in ECs [110]. As FOXO1 and p53 strongly induce endothelial SIPS by cell cycle arrest, Sirtuin-1 maintains the angiogenic properties of PTC ECs by counteracting SIPS [108]. Deletion of endothelial Sirtuin-1 enhanced PTC loss via down-regulation of matrix metalloproteinase (MMP)-14 and activation of Notch1 signaling [111,112]. ECs needed MMP-14 to degrade the extracellular matrix for new microvessel formation [111]. In ECs, Notch1 induced SIPS and cell cycle arrest by elevated PTEN expression [112,113]. Separately, loss of endothelial Sirtuin-1 extensively reduced endothelial glycocalyx [114]. Reduced glycocalyx was shown to lower microvascular perfusion in rodents and humans [115,116,117], suggesting that Sirtuin-1 deficient PTCs lose their perfusion and induce tissue hypoxia following kidney injury. Taken together, endothelial Sirtuin-1 is protective against PTC loss.

### 5.5. Vasohibin

Vasohibin-1 (VASH-1) was initially identified as a novel antiangiogenic factor derived from ECs [118], while Vasohibin-2 (VASH-2) was identified as a VASH-1 homolog and a novel proangiogenic factor [119]. VASH-1 is expressed in glomerular and capillary ECs of the normal kidneys, and kidney injury induces VASH-1 expression in glomerular mesangial cells and inflammatory cells in addition to ECs [120]. Later study found that endothelial VASH-1 increases the expression of superoxide dismutase-2 and the synthesis of Sirtuin-1 in ECs, indicating that endothelial VASH-1 promotes stress tolerance by quenching reactive oxygen species and SIPS [121]. In response to cisplatin-induced kidney injury, VASH-1 deficient mice enhanced loss of renal function, tubular injury, macrophage infiltration, and PTC rarefaction compared with control mice [122]. Kidney injury decreased VASH-1 expression in whole kidneys and accelerated PTC loss [122]. VASH-2 expression is observed in ECs of PTC/vasa recta and cortical/medullary tubules in the normal kidney and is strongly induced in tubular epithelial cells after kidney injury [123]. Following IRI, VASH-2 knockout mice exaggerated loss of renal function, tubular injury, neutrophil infiltration, and PTC rarefaction compared with control mice [124]. VASH-2 expression was intensely increased in damaged kidneys, antagonizing PTC rarefaction. Collectively, VASH protects against PTC loss.

### 5.6. Pericyte-Endothelial Cell Interaction

As mentioned above, endothelial-pericyte crosstalk is critical for angiogenesis and vascular stabilization. This crosstalk involves multiple ligand-receptor interactions including PDGF-B/PDGF receptor-β (PDGFRβ) and Angpt-1/Tie2 [125]. When either PDGFRβ signaling in pericytes or VEGFR2 signaling in ECs was blocked by circulating soluble receptor ectodomains, both PTC rarefaction and fibrosis were remarkably alleviated during CKD progression [126]. This result indicates that (1) bidirectional signaling between pericytes and PTC ECs is necessary to prevent pericyte detachment from PTCs, and (2) kidney injury excessively enhances this bidirectional signaling, resulting in pericyte loss and unstable vasculatures. Once pericytes were detached from PTCs, angiogenesis of functional PTC was disrupted and inefficient, which could be corrected by PDGFRβ or VEGFR-2 blockade [126]. While pericytes synthesized EPO in normal kidneys, myofibroblasts lost the capability of producing EPO, which induced renal anemia and thereby enhanced tissue hypoxia [71,127,128]. Impaired EPO/EPO receptor signaling was shown to cause PTC rarefaction post-IRI [129]. Moreover, tissue hypoxia played a critical role in acceleration of PTC loss after kidney injury [41].

### 5.7. Endothelial Progenitor Cells

Endothelial progenitor cells (EPCs) are defined as non-ECs that are capable of differentiating into ECs. Asahara et al. provided the first evidence that bone marrow-derived circulating EPCs differentiate into mature ECs to form new vessels in vivo [130]. Following this primary publication, numerous studies have shown that EPCs contribute to vascular regeneration in patients and multiple models of tissue injury including AKI and CKD. However, later studies detected only marginal EPC incorporation into the vasculatures in animal models of AKI and CKD [131,132,133]. Furthermore, all new blood vessels in the damaged heart were derived from pre-existing ECs, not from EPC [134]. These results suggest an idea that EPCs stimulate PTC angiogenesis via paracrine mechanisms such as secretion of proangiogenic factors (VEGF-A and Angpt-1) to repair damaged vasculatures [131]. Alternatively, EPCs may have the potential to prevent pericyte detachment from PTCs [135].

### 5.8. Endothelial to Mesenchymal Transition (EndMT)

The concept of EndMT is that endothelial cells are capable of differentiating into mesenchymal cells (fibroblasts and myofibroblasts) under certain conditions. The initial study demonstrated that 30–50% of myofibroblasts are derived from ECs during kidney injury [136]. However, the later study revealed that only 10% of myofibroblasts emerged via EndMT following kidney injury [137]. Thus, renal ECs mainly disappear without becoming fibroblasts in damaged kidneys. Many studies exploring the role of EndMT in renal fibrosis have used vascular endothelial (VE)-cadherin-Cre (Cdh5-Cre) mice and Tie2-Cre mice for linage tracing or deletion of specific factors in ECs. However, VE-cadherin and Tie2 have been shown to be non-specific for ECs and were expressed in hematopoietic cells during development, because of endothelial origin of hematopoietic stem cells [138]. The lack of a specific marker for ECs for lineage tracing makes it difficult to interpret these studies properly. As no evidence of EndMT was detected in cardiac fibrosis following injury [139], further study is necessary to determine the minor contribution of EndMT to PTC rarefaction.

## 6. Assessment of PTC Loss or Its Surrogate Marker, Tissue Hypoxia

### 6.1. Histological Assessment

Histological evaluation is the popular method to assess PTC density. Many researchers have immunostained kidney sections with EC marker(s) and measured PTC density by a grid method (% of EC marker-positive grids among total grids) or by an area fraction method (% of EC marker-positive area among total area). However, these methods may overestimate PTC density, because ECs of clogged or collapsed capillaries with micro-embolisms are still stained and positive for EC marker(s), even if PTCs totally lose their perfusion. Kramann et al. injected the fluorescence microbead (0.02-μm diameter)-agarose mixture into the beating heart to visualize actual capillary lumen in mouse kidneys post-IRI [42]. They found that total perfused PTC cross-sectional area, PTC number, individual capillary cross-sectional area, and individual capillary perimeter are significantly decreased in damaged kidneys compared with sham-operated control kidneys 8 weeks post-severe IRI. Moreover, these factors of PTC density were inversely correlated with renal function assessed by BUN. For more accurate assessment of PTC density, Babickova and colleagues proposed a normalization of PTC counts to the number of adjacent tubular segments, because the number of tubules differed between rodent CKD models [48]. For example, the number of tubules per area remained constant (with the appearance of dilated and atrophic tubules), but the number of PTCs per field decreased during CKD progression in UUO model, whereas the number of PTCs was not significantly lower, but the number of tubules was higher (given the many atrophic tubules and only very few dilated tubules) in IRI model. In Alport mice, both the number of PTC and tubules reduced, but loss of PTC was more significant [48]. Further study is necessary to determine which method is most optimized for the assessment of PTC density in the course of CKD progression. Although a histological assessment of PTC density by immunostaining is the most popular method for animal models of CKD, it could not be used for follow-up of CKD patients because tissue sampling is invasive.

### 6.2. Micro-Computed Tomography (Micro-CT)

Micro-computed tomography (Micro-CT) provided the ability to create high resolution images of renal microvasculature such as arterioles (their diameter is bigger than 20 μm) [49]. As the diameter of PTC is typically 5–10 μm [22], Micro-CT does not have sufficient power to visualize the smallest size of capillaries. However, the recently developed microangio-CT method was utilized to visualize capillaries with high resolution [140]. This new method used a new polymerizing contrast agent and successfully visualized PTCs in the mouse, showing that created PTC images are comparable to immunostaining images [140]. Although microangio-CT could assess PTC density much faster than histology-based methods, further study is necessary for its general use.

### 6.3. Renal Resistive Index

Renal resistive index (RRI) is noninvasively measured by renal Doppler ultrasonography and is calculated with the following formula: (peak systolic velocity—end diastolic velocity)/peak systolic velocity. RRI not only reflects changes in intrarenal perfusion but is also related to systemic hemodynamics and the presence of subclinical microvascular atherosclerosis in the kidney. In 30 patients with CKD, RRI positively correlated with systolic blood pressure, interstitial fibrosis, and arteriosclerosis, and negatively correlated with PTC density and creatinine clearance (renal function) [141]. A multivariate analysis demonstrated that PTC loss emerged as one of independent variables associated with RRI elevation. As RRI could be measured repeatedly, RRI may be a feasible method to periodically assess PTC rarefaction in CKD patients.

### 6.4. Endothelial Micro-Particles

Endothelial micro-particles (EMPs) are extracellular vesicles that are shed by damaged (activated) ECs. In urine samples collected from patients with essential and renovascular hypertension, the levels of EMPs, which were positive for plasmalemmal-vesicle-associated protein (PL-VAP) and negative for CD31 as well as CD144 (VE-cadherin), significantly increased compared with those in urine samples from healthy subjects [142]. In the kidney, PL-VAP (PV-1) was abundantly expressed in diaphragms of endothelial fenestrae of PTCs and AVRs (Figure 3A), while it was undetectable in arterial and glomerular ECs [29,143]. Urine PL-VAP+ EMP levels, but not circulating EMPs, were inversely correlated with histologically assessed PTC density, GFR, and renal blood flow [142], suggesting that increased levels of urine EMPs reflect PTC rarefaction. However, glomerular injury promoted PL-VAP expression in glomerular ECs [144,145], and PL-VAP was abundantly expressed in ECs of other organs [29]. These issues must be cleared before its clinical use for PTC assessment.

### 6.5. BOLD-MRI

One of the major outcomes of PTC rarefaction is tissue hypoxia. Blood oxygen level-dependent (BOLD) magnetic resonance imaging (MRI) could be an indirect and non-invasive assessment of PTC rarefaction by evaluating renal hypoxia. BOLD-MRI used the principle that magnetic properties of hemoglobin depend on its oxygenated status. Increased local deoxyhemoglobin levels caused decreased T2* (tissue parameter and expressed in sec) or increased R2* (decay rate, defined as 1/T2* and expressed in 1/sec) [146]. CKD patients showed significantly decreased T2* value in their kidneys compared with healthy controls [147]. Decline in GFR correlated with increased R2* value of the cortical layers [148], suggesting that cortical hypoxia deteriorates renal function. BOLD-MRI does not require the administration of contrast agents (possibly nephrotoxic) and could be repeated multiple times without side effects.

## 7. Therapy to Mitigate PTC Rarefaction

### 7.1. Anti-Hypertensive Drugs

Hypertensive kidney diseases are one of the major causes of CKD. Several anti-hypertensive drugs were demonstrated to counteract PTC loss (or microvascular loss) in experimental animal models. In the rat model with age-dependent progressive kidney diseases, angiotensin-converting enzyme (ACE) inhibitor or angiotensin II receptor blocker (ARB) antagonized PTC rarefaction [149]. These drugs inhibited EC apoptosis and augmented EC proliferation, though the control of blood pressure was not reported [149]. In the rat hypertensive model with angiotensin II infusion, ACE inhibitor and ARB antagonized PTC rarefaction independent of blood pressure lowering effect [150]. Moreover, ARB preserved PTC perfusion and ameliorated tissue hypoxia in damaged kidneys [151]. In the rat uninephrectomy model with saline and mineralocorticoid infusion, spironolactone (SPL) counteracted hypertension, loss of creatinine clearance (renal function), and PTC loss [6]. SPL inhibited endothelial apoptosis and TSP-1 expression although it did not affect endothelial proliferation. In the pig model with renovascular diseases (renal artery stenosis), endothelin-A receptor (ET-A) blocker, but not ET-B blocker, ameliorated hypertension, reduction in GFR, and loss of microvasculature [152]. As the diameter of microvasculature was defined as less than 200 μm in that study, it mainly consisted of arterioles and venules instead of PTCs [152]. Consistent with this finding, long term use of ET-A blocker retarded CKD progression in patients with type 2 diabetes in the international trial, though water retention was its major and serious side effect [153]. These anti-hypertensive drugs could be effective to block PTC rarefaction during CKD progression.

### 7.2. Sodium Glucose Cotransporter Inhibitor for Diabetic Nephropathy

PTC rarefaction was identified in diabetic nephropathy, a leading cause of CKD [4]. In large clinical trials in patients with type 2 diabetes, inhibitors of sodium-glucose cotransporter 2 (SGLT2) significantly prevented the progression of diabetic nephropathy to ESRD [154,155]. SGLT2 inhibitor protected tubular epithelial cells from the toxicity of chronic hyperglycemia, resulting in preservation of tubular VEGF-A synthesis [156]. In addition to blood glucose lowering effect, SGLT2 inhibitor maintained tubular VEGF-A expression following IRI, antagonizing PTC rarefaction and fibrosis [156]. Interestingly SGLT2 inhibitor retarded CKD progression in patients without diabetes [157].

### 7.3. Tie2 Activator

While kidney injury compromised Tie2 activity by increased VE-PTP expression, chemical or genetic Tie2 activation sustained PTC integrity following injury, preserving GFR [96,158]. As mentioned above, VE-PTP inhibition maintained Tie2 activation (Tie2 phosphorylation) independent of Angpt-1 or Angpt-2. In clinical trials of patients with diabetic macular edema, subcutaneous injections of VE-PTP phosphatase inhibitor (AKB-9778) were well-tolerated for 12 weeks [159]. In some of such diabetic patients, AKB-9778 improved albuminuria, suggesting that AKB-9779 is a renal protective agent.

### 7.4. Nicotinamide (NAM)

Although Sirtuin-1 activators such as resveratrol have been demonstrated to successfully treat kidney injury in animal models [160], there have been no clinical trials to assess Sirtuin-1 activators for patients with kidney diseases [108]. NAD+ is required for Sirtuin-1 function. Supplementation with nicotinamide mononucleotide (NMN), an NAD+ precursor, protected mice from cisplatin-induced AKI via restored renal Sirtuin-1 activity [161]. Since AKI lowered renal NAD+ level, patients were orally administered with nicotinamide (NAM) for 3 days before and after cardiac surgery to assess whether NAM could block AKI development [162]. NAM is converted to NAD+ through the intermediate NMN. NAM treatment significantly increased circulating NAD+ level and prevented perioperative AKI compared with placebo, suggesting that increasing NAD+ level is protective against PTC loss and kidney injury by enhancing Sirtuin-1 activity.

### 7.5. PHD Inhibitor

PHD inhibitor robustly stabilizes HIF-1α and HIF-2α, which dimerize with HIF-1β and increase transcription of HIF target genes including EPO and VEGF-A. Since tissue hypoxia worsens PTC loss, HIF stabilizers are expected to improve PTC rarefaction by correcting renal anemia. In two clinical trials with non-dialysis and dialysis patients, PHD inhibitors (roxadustat) were tolerated well for 26 weeks and effectively improved renal anemia [163,164]. Moreover, PHD inhibitors effectively promoted erythropoiesis by lowering circulating hepcidin level in treated patients. In the study of knockout mice, HIF-2α promoted hepatic and renal EPO production, which inhibited hepatic hepcidin expression [165]. Reduced hepcidin level increases dietary iron absorption and iron release from macrophages, facilitating erythropoiesis. PHD inhibitors also increased VEGF-A level in CKD animal models, which may favor PTC preservation [166].

### 7.6. Nintedanib

Targeting endothelial-pericyte cross-talk may provide a novel therapeutic opportunity to prevent PTC loss and fibrosis. In the clinical setting, Richeldi et al. treated patients with idiopathic pulmonary fibrosis (IPF) for 1 year with nintedanib (formerly known as BIBF 1120), an intracellular inhibitor that can block multiple tyrosine kinase receptors such as PDGFRβ and VEGFR-2 [167]. Nintedanib significantly retarded IPF progression, suggesting a beneficial effect of the drug on tissue fibrosis. In an animal model of systemic sclerosis, nintedanib was shown to reduce capillary rarefaction and tissue fibrosis [168]. Although the most frequent adverse effect of nintedanib was diarrhea, more than 95% of patients tolerated the drug intake during the study period [167]. Nintedanib may have a potential to normalize endothelial-pericyte crosstalk and prevent pericyte detachment as well as PTC rarefaction during CKD progression.

## 8. Capillary Rarefaction in Other Organs

This review focused on the roles of PTC rarefaction in the progress of CKD. However, capillary rarefaction has been found to be correlated with organ dysfunction in heart [169], lung [170,171], skin [172], muscle [173], retina [174], and brain [175], suggesting that capillary rarefaction could be an important and universal component to determine future declines in the function of various organs.

## 9. Conclusions

AKI and CKD universally cause PTC rarefaction in the kidney. As glomerular capillaries and PTCs/DVRs are interconnected without collateral vessels, PTC loss causes the loss of GFR (the loss of kidney function) and vice versa. Recent studies have identified novel mechanisms of PTC rarefaction and indicated that PTC rarefaction is not only a prominent histological characteristic of CKD but also a central driving force of CKD progression. As we currently obtain new tools to assess PTC density and multiple drugs to mitigate PTC rarefaction in patients, PTC rarefaction would become a practical therapeutic target to halt the progression of CKD.

## Abbreviations

CKD	chronic kidney disease
PTC	peritubular capillary
ESRD	end-stage renal disease
DVR	descending vasa recta
AVR	ascending vasa recta
EC	endothelial cell
GFR	glomerular filtration ratio
VEGF-A	vascular endothelial growth factor-A
VEGFR2	vascular endothelial growth factor receptor-2
BUN	blood urea nitrogen
Angpt-1	angiopoietin-1
TSP-1	thrombospondin-1
TNF-α	tumor necrosis factor-α
IL-1β	interleukin-1β
UUO	unilateral ureteral obstruction
IRI	ischemia reperfusion injury
PI3K	phosphoinositide-3-kinase
FOXO1	forkhead box O-1
PTEN	phosphatase and tensin homolog
HIF	hypoxia-inducible factor
FoxD1	forkhead box D-1
TGF-β	transforming growth factor-β
EPO	erythropoietin
PDGF-B	platelet-derived growth factor-B
AKI	acute kidney injury
VE-PTP	vascular endothelial-protein tyrosine phosphatase
PHD	prolyl hydroxylase domain-containing protein
NAD	nicotinamide adenine dinucleotide
SIPS	stress-induced premature senescence
MMP	matrix metalloproteinase
VASH	vasohibin
PDGFRβ	platelet-derived growth factor receptor-β
EPC	endothelial progenitor cell
EndMT	endothelial to mesenchymal transition
RRI	renal resistive index
EMP	endothelial microparticles
PL-VAP	plasmalemmal-vesicle-associated protein
BOLD-MRI	blood oxygen level-dependent-magnetic resonance imaging
ACE	angiotensin-converting enzyme
ARB	angiotensin II receptor blocker
SPL	spironolactone
ET-A	endothelin-A receptor
SGLT2	sodium glucose cotransporter 2
NMN	nicotinamide mononucleotide
NAM	nicotinamide
IPF	idiopathic pulmonary fibrosis
